# Opportunities and Challenges of Visual Large Language Models in Imaging Diagnostics: Lessons from Brain Metastasis Detection in Clinical MRI

**DOI:** 10.3390/diagnostics16050749

**Published:** 2026-03-03

**Authors:** Christian Nelles, Nour Abou Zeid, Robert Terzis, Andra-Iza Iuga, Lukas Görtz, Marvin A. Spurek, David Maintz, Simon Lennartz, Jonathan Kottlors

**Affiliations:** Institute for Diagnostic and Interventional Radiology, Faculty of Medicine, University Hospital Cologne, University of Cologne, 50937 Cologne, Germany; nour.abou-zeid@uk-koeln.de (N.A.Z.); robert.terzis@uk-koeln.de (R.T.); andra.iuga@uk-koeln.de (A.-I.I.); lukas.goertz@uk-koeln.de (L.G.); marvin.spurek@uk-koeln.de (M.A.S.); simon.lennartz@uk-koeln.de (S.L.); jonathan.kottlors@uk-koeln.de (J.K.)

**Keywords:** visual large language models, brain metastases, Magnetic Resonance Imaging, diagnostic accuracy

## Abstract

**Background/Objectives**: To evaluate the diagnostic accuracy of two visual large language models (vLLMs), GPT-4o (OpenAI) and Claude Sonnet 3.5 (Anthropic), for detecting brain metastases in routine MRI using combined imaging and textual input. **Methods**: This retrospective study included 31 patients with and 46 without brain metastases with underlying melanoma (*n* = 24), lung cancer (*n* = 23), breast cancer (*n* = 17), or renal cell carcinoma (*n* = 13). In total, 100 MRI examinations (50 with, 50 without metastases) were provided to both vLLMs using a single representative slice per sequence, together with clinical history and the referring question. The generated free-text reports were evaluated for detection accuracy, overdiagnosis, correct sequence recognition, anatomical localization, lesion laterality, and lesion size estimation. **Results**: Both vLLMs showed perfect sensitivity (100% for both) but very low specificity (GPT-4o: 8%, Sonnet 3.5: 4%; *p* = 0.625), resulting in low diagnostic accuracy (GPT-4o: 54%, Sonnet 3.5: 52%; *p* = 0.625). Sequence identification was highly accurate in both models, with GPT-4o performing significantly better (100% vs. 93%; *p* < 0.05). Identification of the anatomical brain region (70% vs. 72%; *p* = 1.00) and lesion laterality (62% vs. 76%; *p* = 0.189) was comparable. Both models hallucinated additional lesions in 12% of cases. Lesion size measurements showed no significant differences between the models or in comparison with the radiologist. **Conclusions**: GPT-4o and Claude Sonnet 3.5 can generate radiological reports and detect brain metastases with excellent sensitivity, but their very low specificity, frequent hallucinations, and limited spatial reliability currently preclude clinical application. Future work should address how the balance between visual and textual input influences diagnostic behavior in vLLMs.

## 1. Introduction

Brain metastases are a common complication in patients with advanced malignancies, with a reported lifetime prevalence of 10–30% among cancer patients [1,2]. They are an important cause of morbidity and mortality and can significantly impact the course of treatment [3,4]. This illustrates the importance of radiological screening and early detection of brain metastases, for which MRI is the imaging modality of choice [5,6]. The incidence of brain metastases is expected to rise due to improvements in systemic cancer therapy and the resulting improved overall survival [7,8,9]. This also leads to a steadily growing workload for radiologists. Given the increasing imaging workload, the development and implementation of artificial intelligence (AI) technologies that can support radiologists is crucial. Ideally, such a solution should be able to take into account the medical history of a patient, as it has a fundamental influence on the pretest probability of brain metastases.

In the past, convolutional neural networks (CNNs), a type of deep learning architecture designed for image analysis and interpretation, have become an integral part of AI applications in radiology and are currently being employed for a wide range of tasks, especially for lesion detection, disease characterization and image segmentation [10,11,12,13]. While they are proficient in performing specific image recognition tasks for which they were trained, they have certain limitations: firstly, they do not take into account non-visual contextual information such as the medical history of a patient; secondly, they do not incorporate longitudinal imaging data and cannot interpret an image in a broader context and thirdly, they are dependent on the quality of the dataset which they were trained on [11].

In contrast, large language models (LLMs) like OpenAI’s GPT-series are deep learning models based on the transformer architecture that excel in a broad range of natural language processing tasks, such as text generation or summarization, and in understanding context [14]. With their ability to analyze large bodies of text and interpret them in a broader context, they have shown great potential for application in radiology in preliminary studies, e.g., for summarization of important findings or suggestion of differential diagnoses on the basis of radiological reports [14,15,16].

Recently, visual large language models (vLLMs) have emerged and can analyze both textual and visual data by combining vision transformers (ViTs) and conventional LLMs. ViTs treat an image as a sequence of patches and apply a transformer model to these patches, allowing them to recognize spatial relationships and hierarchies in the image data. This approach offers an alternative to image analysis by CNNs, providing a better contextual interpretation similar to how traditional LLMs process text and also the ability to analyze more complex and varying image datasets [17,18].

With the potential ability to integrate visual and textual data, vLLMs have the theoretical potential to interpret medical images and to create a radiological report by incorporating medical history and prior imaging studies and therefore to fundamentally transform the radiological workflow. Preliminary studies have shown promising results for the ability of vLLMs to interpret non-radiological medical image data [19,20]. In contrast, initial studies on the interpretation of radiological images, focusing primarily on solving clinical vignette questions, yielded mixed results [20,21,22,23]. In particular, only isolated studies investigating the application of vLLMs in image interpretation of routine clinical cases exist so far and suggest a lack of suitability for this purpose, citing fabrication of findings as one of the problems [22,24].

The aim of this study was to conduct an initial proof-of-concept evaluation of two vLLMs for the detection of brain metastases of different tumors in cranial MRI and for their ability to combine imaging and textual information for the generation of radiological reports. We deliberately chose this task, as metastasis detection in cMRI offers a comparatively standardized setting with limited sequence requirements and high lesion-to-background contrast, making it a suitable starting point for assessing the feasibility of applying vLLMs to clinical imaging data.

## 2. Materials and Methods

### 2.1. Study Cohort

The study was approved by the ethics committee (approval number: 24-1252-retro), and informed consent was waived due to its retrospective design. The study was conducted in accordance with the Declaration of Helsinki. To identify patients eligible for study inclusion, the radiological information and picture archiving and communication system were screened to identify patients meeting the following criteria:

Patients ≥ 18 years who were diagnosed with either non-small cell lung cancer (NSCLC), malignant melanoma, breast cancer or renal cell carcinoma and who did not suffer from a secondary malignancy.
Received a clinically indicated MRI of the brain between 1 January 2017 and 1 August 2024 during routine cancer follow-up.
(a) For the metastatic group, the presence of brain metastases had to be mentioned in the radiological report. Metastases had to be confirmed either by histopathology or by the prior or follow-up examinations with an increase in size or a shrinkage during treatment.
(b) For the non-metastatic group, lack of brain metastases had to be mentioned in the radiological report, which was confirmed in a ground truth annotation.

**Figure 1:**
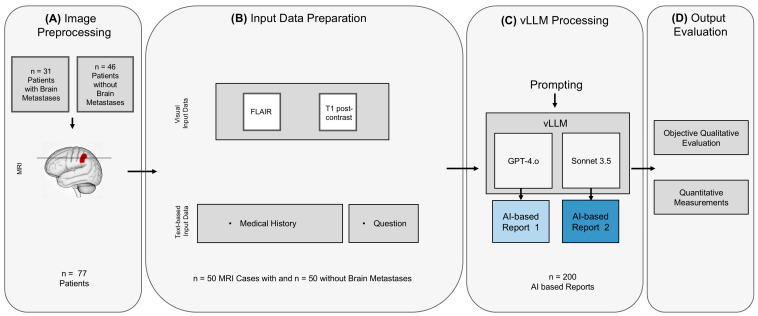
The diagram presents a flowchart illustrating the process of the study involving the evaluation of brain metastases using AI-driven analysis and report generation: Image preprocessing (**A**): MRI data from patients with and without brain metastases were retrospectively selected. For patients with metastases, a pathology-capturing axial slice and for patients without metastases, a random slice was defined. In total, *n* = 50 MRI cases with and *n* = 50 without metastases were exported. Input data preparation (**B**): The visual input comprised one representative slice from two MRI sequences each (FLAIR and contrast-enhanced T1-weighted images). The textual input included structured metadata such as the examination date, relevant clinical history, and the clinical indication, including information on prior or ongoing treatments. The combined use of imaging and clinical text data was intended to reflect a realistic diagnostic setting and to enable comprehensive multimodal analysis by the AI models. vLLM processing (**C**): The prepared multimodal input was provided to two visual large language models (GPT-4o and Sonnet 3.5). Both models were instructed to analyze the MRI images in conjunction with the accompanying clinical information and to produce structured radiological reports integrating both sources of information. Output evaluation (**D**): The generated reports were subsequently reviewed by an experienced radiologist. Evaluation included qualitative assessment of report accuracy and completeness, as well as quantitative analysis such as comparison of reported and reference tumor size measurements. Abbreviations: FLAIR—fluid-attenuated inversion recovery; vLLM—visual large language model; AI—artificial intelligence.

From these patient groups, *n* = 50 consecutive MRI examinations with and *n* = 50 without metastases were selected for further analysis (*n* = 15 with melanoma, *n* = 15 with NSCLC, *n* = 10 with breast cancer and *n* = 10 with renal cell carcinoma, respectively). The study workflow consisted of four main steps: image preprocessing, input data preparation, vLLM processing and output evaluation (Figure 1).

### 2.2. Image Acquisition, Preprocessing and Input Data

All patients were examined at a magnetic field strength of 3 Tesla. MRI protocols included 3D Fluid-attenuated inversion recovery (FLAIR), susceptibility-weighted and contrast-enhanced, fat-saturated 3D T1w sequences.

**Figure 2:**
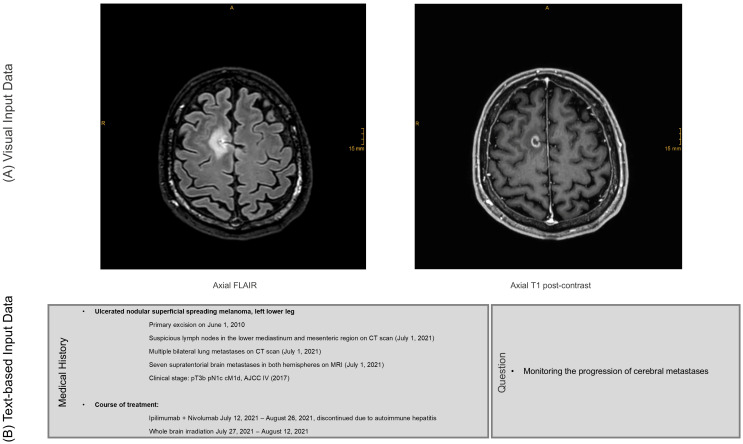
Input Data for AI-Generated Radiology Reports. (**A**) Visual input: MRI images consisting of single-slice axial FLAIR and contrast-enhanced T1-weighted sequences, including a visible scale reference to allow for size estimation. (**B**) Textual input: Clinical background information, including the patient’s medical history, relevant clinical details and the referring physician’s question. Both the imaging data and clinical information were provided to the AI models to enable report generation under conditions resembling routine clinical practice. Abbreviations: FLAIR—fluid-attenuated inversion recovery; AI—artificial intelligence.

For each case, two 2D images from the corresponding MRI examination were extracted, including a FLAIR image and a T1-weighted post gadolinium contrast image of the same slice. In patients with metastases, the slice was selected in such a way that the lesion was visible at its maximum extent and that no other lesion was depicted in the same slice. In cases without metastases, a random slice was selected. The slices were exported in JPEG (Joint Photographic Experts Group) format without annotations but including a visible scale bar (Figure 2A). Additionally, the patient’s clinical history and the specific clinical question for the imaging study were included in text form (Figure 2B). This experimental design of a single-slice evaluation was chosen because the models currently do not support analysis of complete volumetric datasets.

### 2.3. vLLM Processing

#### 2.3.1. Visual Large Language Models

The visual large language models evaluated in this study were the generative pre-trained transformer model GPT-4o (OpenAI, San Francisco, CA, USA) and Claude Sonnet 3.5 (Anthropic, San Francisco, CA, USA), both equipped with integrated vision capabilities. The MRI images, together with the corresponding clinical history and referring question, were provided to each model through their respective online interfaces. Each case was submitted in an independent conversation to ensure that model outputs were not influenced by prior interactions.

#### 2.3.2. Prompt Engineering and AI-Generated Reports

The prompt was developed and optimized through an iterative process consisting of ten refinement cycles using MRI data from five patients who were not included in the study cohort. This procedure allowed systematic evaluation and improvement of prompt clarity and specificity. Prompt development was conducted by two radiologists (JK and SL) who were not involved in the subsequent expert assessment. The finalized prompt combined open-ended instructions with specific guidance to encourage the generation of structured, narrative radiology reports. Identical input data and the same prompt were used for both models to ensure methodological consistency. The final prompt was as follows:

• Imagine you are a radiologist. The two images are sequences from the same examination.
• Please identify the sequences. Then, create a report and an assessment in which you address the overall question.
• Please also take the medical history into account. Indicate whether any pathological findings are visible. If so, indicate the size of the lesion (the contrast-enhancing portion in two axes) and indicate where the lesion is localized (anatomical brain region and side).

The aiRR was extracted for each case (Figure S1) and copied into specific evaluation forms.

### 2.4. Output Evaluation

For evaluation, an output consisting of the two axial slices, the patient’s clinical history, the specific clinical question and the AI-generated radiology report (aiRR) in text form was presented (Figure 2). Evaluation was performed by a board-certified radiologist with 7 years of experience in brain MRI imaging. The aiRR were evaluated using the following objective, binary criteria:

• Both MRI sequences were correctly identified;
• Presence or absence of a pathological lesion in the presented images was correctly identified;
• Anatomical region of the lesion, if present, was correctly identified (e.g., frontal lobe);
• Side of the lesion, if present, was correctly identified;
• In case of an identified lesion, whether additional lesions were wrongfully detected or not.

Additionally, reported quantitative measurements of the contrast-enhancing part of the lesions in long and short axis in the aiRR were compared to measurements by the radiologist in the picture archiving and communication system. The measurements by the radiologist were taken in the same slice of the 3D T1w post-contrast sequence, which was presented to the vLLMs.

### 2.5. Statistical Methods

All statistical analyses were performed using Python 3.12 (Python Software Foundation, Wilmington, DE, USA). The Wilson method was used to calculate 95% confidence intervals for binomially distributed data. Paired binomial data were compared with McNemar’s exact test. Paired interval data were compared with paired *t*-tests and adjusted for multiple comparisons with Bonferroni correction. *p* < 0.05 indicated statistical significance.

## 3. Results

### Study Cohort

A total of 77 patients (mean age 58.8 ± 12.7 years), 32 men (mean age 57.8 ± 15.1 years) and 45 women (mean age 59.7 ± 11.4 years), were included in the study: *n* = 31 with brain metastases (*n* = 9 with melanoma, *n* = 8 with NSCLC, *n* = 7 with breast cancer and *n* = 7 with renal cell carcinoma) and *n* = 46 without brain metastases (*n* = 15 with melanoma, *n* = 15 with NSCLC, *n* = 10 with breast cancer and *n* = 6 with renal cell carcinoma). This corresponded to *n* = 50 brain MRI examinations with and *n* = 50 without metastases (*n* = 15 with melanoma, *n* = 15 with NSCLC, *n* = 10 with breast cancer and *n* = 10 with renal cell carcinoma, respectively). Basic demographic information is summarized in Table 1.

*a.*  *Evaluation of the AI-generated reports*

**Figure 3:**
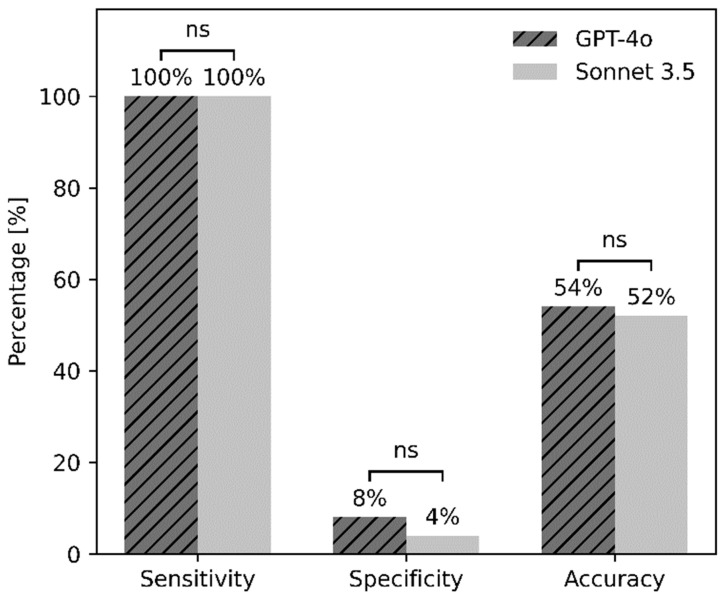
Sensitivity, specificity and diagnostic accuracy of GPT-4o and Sonnet 3.5 for the detection of brain metastases. Abbreviations: ns—not significant.

Both GPT-4o (G) and Sonnet 3.5 (C) demonstrated perfect sensitivity in detecting brain metastases, with a sensitivity of 100% (95% CI: 92.9–100%) in both models (50/50 cases, *p* = 1.00). Specificity was low in both cases, with G achieving 8% (95% CI: 3.2–18.8%, 4/50) and C achieving 4% (95% CI: 1.1–13.5%, 2/50), showing no significant difference (*p* = 0.625). The resulting diagnostic accuracy was 54% for G and 52% for C, with no statistically significant difference (*p* = 0.625). Figure 3 shows the sensitivity, specificity and diagnostic accuracy of both models.

G correctly identified the MRI sequences in all cases (100%, 95% CI: 96.3–100%, 100/100), while C correctly identified the sequences in 93% of cases (95% CI: 86.3–96.6%, 93/100), with a statistically significant difference in favor of G (*p* < 0.05).

Correct identification of the anatomical brain region was comparable between the two models, with an accuracy of 70% for G (95% CI: 56.2–80.9%, 35/50), while C achieved 72% (95% CI: 58.3–82.5%, 36/50), without a significant difference (*p* = 1.00). When evaluating the correct side of the brain, C achieved a higher accuracy with 76% (95% CI: 62.6–85.7%, 38/50) compared to 62% for G (95% CI: 48.2–74.1%, 31/50), which was not statistically significant (*p* = 0.189). For over-detection of metastases in images with a depicted single metastasis, both models wrongfully reported additional lesions in 12% of cases (95% CI: 5.6–23.8%, 6/50; *p* = 1.00). Table 2 summarizes the results of the objective qualitative evaluation.

*b.*  *Lesion size estimation*

**Figure 4:**
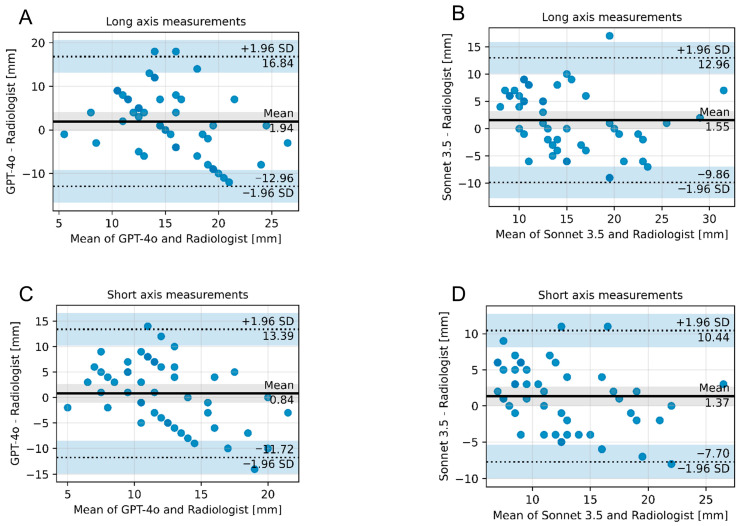
Bland–Altman plots illustrating the correspondence of size measurements between the vLLM models and the radiologist. (**A**) Long axis measurement, GPT-4o vs. radiologist. (**B**) Long axis measurement, Sonnet 3.5 vs. radiologist. (**C**) Short axis measurement, GPT-4o vs. radiologist. (**D**) Short axis measurement, Sonnet 3.5 vs. radiologist. Abbreviations: SD—standard deviation.

The mean estimated diameter of the lesions by G was 16.3 mm (SD 4.3 mm) for the long axis and 12.7 mm (SD 3.6 mm) for the short axis and by C was 16.0 mm for the long axis (SD 5.4 mm) and 13.2 mm (SD 4.4 mm) for the short axis. In comparison, the mean size measured by the radiologist was slightly smaller, with 14.4 mm (SD 6.9 mm) in the long axis and 11.7 mm (SD 5.9 mm) in the short axis. However, there were no statistically significant differences between the measurements of the radiologist and both models and between the two models themselves (long axis: G vs. C: *p* = 1.00; G vs. radiologist: *p* = 0.232; C vs. radiologist: *p* = 0.205; short axis: G vs. C: *p* = 1.00; G vs. radiologist: *p* = 1.00; C vs. radiologist: *p* = 0.132). Figure 4 illustrates the correspondence of measurements between the vLLM models and the radiologist.

## 4. Discussion

In this study, we evaluated two vLLMs, GPT-4o and Claude Sonnet 3.5, for the detection of brain metastases on clinical MRI and the generation of radiological reports by integrating medical history with visual data. This task was chosen to simulate a realistic clinical scenario, where image interpretation must be embedded within the patient’s clinical context.

Both models achieved perfect sensitivity (100%) but showed very poor specificity, leading to frequent overdiagnosis of metastases. This trade-off reflects the well-known problem of hallucinations in LLMs and represents a central limitation in their current stage of development [14]. From a clinical perspective, the extremely high false-positive rate is particularly concerning, as it could lead to unnecessary follow-up imaging, additional diagnostic procedures, patient anxiety and increased healthcare costs. In this context, diagnostic accuracy and sensitivity alone are insufficient to assess clinical utility. A diagnostically useful assistive system must achieve an appropriate balance between sensitivity and specificity to avoid overdiagnosis and potential harm. Therefore, despite the high sensitivity observed in this study, the current specificity levels represent the primary barrier to clinical applicability. Both models also correctly identified MR sequences in most cases, with G performing slightly better, and provided lesion size estimates comparable to those of the radiologist. However, anatomical localization was less consistent, which may further limit clinical applicability in scenarios requiring precise spatial information, such as surgical planning or treatment monitoring.

The extremely low specificity observed in our study likely reflects inherent characteristics of generative multimodal models rather than a single isolated factor. vLLMs are designed to produce coherent and contextually plausible outputs rather than conservative diagnostic decisions. In the presence of a clinical context indicating malignancy, this may result in a bias toward confirming expected findings. Furthermore, multimodal integration itself may introduce bias, as textual clinical information can disproportionately influence interpretation and potentially override ambiguous visual features. Limited visual input may further increase diagnostic uncertainty and encourage overinclusive interpretations. These mechanisms highlight fundamental challenges in applying generative multimodal models to diagnostic imaging tasks requiring high specificity and reliability. Future improvements to vLLMs to address this issue may include domain-specific fine-tuning on radiological datasets, calibration strategies to reduce false-positive outputs, improved prompt design, and the ability to analyze full volumetric imaging datasets.

Due to their ability to analyze large volumes of text within a broader clinical context, traditional LLMs have shown considerable potential in radiology, for example, in summarizing findings or suggesting differential diagnoses [14,15,16]. In contrast, research on vLLMs in radiology is still limited. Existing studies have mainly examined performance in case vignettes or exam-style questions rather than clinical routine [21,23,25,26,27,28]. Reported diagnostic accuracy ranged from comparable or superior to physicians [23,28] to insufficient for patient care [26,27], with hallucinations frequently emphasized as a major limitation.

Only a few studies have addressed vLLM use in clinical routine. Recently, Huppertz et al. [24] analyzed the ability of GPT-4V to detect pathologies in 206 images from different imaging modalities of clinical routine cases with and without additional textual information about the clinical context. Diagnostic accuracy was low without context (8.3%) and improved with context (29.1%), though hallucinations increased, often driven by the textual information. This aligns with our findings of low specificity, frequent false positives in metastasis-free cases, and additional lesions being falsely identified in 12% of true-positive cases. These results suggest an overreliance of vLLMs on textual input compared to imaging data.

Brin et al. [22] investigated the diagnostic accuracy of GPT-4V on 230 emergency medicine images from different modalities without providing textual information about the clinical context and likewise found low accuracy (35.2%) and high hallucination rates (46.8%). Interestingly, modality recognition was perfect (100%) and anatomical localization was accurate in 87.1%. This is comparable to our results, where MRI sequences were identified in 100% and 93% of cases by G and C, and anatomical regions in 70% and 72%, respectively. These findings indicate that vLLMs may be more reliable in modality and anatomy recognition than in diagnostic accuracy.

Prior to the rise of vLLMs, the use of CNNs for automated brain metastasis detection in MRI had already been extensively studied. Compared with conventional CNN approaches, which have achieved sensitivities around 89% and relatively low false-positive rates in brain metastasis detection [29,30,31,32], the vLLMs evaluated in our study showed substantially lower specificity. This is not unexpected, as CNNs are optimized for large imaging datasets, while vLLMs are designed for multimodal integration and broader contextual reasoning. It must also be noted that currently, only individual images can be presented to vLLMs, while CNNs analyze entire data sets. Nevertheless, this highlights that vLLMs are not yet competitive with task-specific deep learning models for metastasis detection.

Apart from the inherent limitations of a retrospective monocenter study design, there are other limitations to our study that need to be addressed. First, the experimental design was based on a single-slice-per-sequence approach rather than full volumetric MRI datasets. This differs fundamentally from clinical routine, where radiologists evaluate complete multi-sequence datasets across multiple slices. Consequently, the models operated with substantially reduced spatial and contextual information, which may have increased diagnostic uncertainty and contributed to false-positive findings and localization errors. Therefore, our findings should be interpreted as a proof-of-concept evaluation under constrained experimental conditions rather than a simulation of routine clinical MRI interpretation.

Second, only one prompt formulation was used, and the temporal consistency of model responses was not assessed. Prompt design may significantly influence model output. The instruction to generate a radiological report and explicitly evaluate for pathological findings may have encouraged abnormality detection and contributed to the high false-positive rate. Generative models are known to be sensitive to prompt framing, and even subtle linguistic differences may influence diagnostic behavior. Future studies should systematically evaluate the effect of prompt design, including more neutral formulations.

Third, visual and textual inputs were always provided together, preventing independent assessment of their respective contributions to diagnostic performance. This limitation is particularly relevant because the clinical history consistently included a known malignancy, which may have biased the models toward metastasis detection by introducing a strong contextual prior. vLLMs are designed to integrate visual and textual information, but such contextual cues may disproportionately influence model outputs. Although this effect cannot be definitively confirmed within the scope of our analysis, it highlights the important question of how the balance between textual and visual input shapes the diagnostic behavior of vLLMs. Addressing this issue will be critical for improving their reliability and clinical applicability. Future studies should therefore systematically compare image-only, text-only, and combined multimodal input conditions to better characterize modality interactions, quantify potential contextual bias, and identify strategies to improve diagnostic specificity.

Beyond technical performance, the deployment of multimodal large language models in clinical settings raises important ethical and patient safety considerations. The high false-positive rates observed in our study illustrate the potential risk of harm if such systems were used without appropriate oversight. Erroneous findings could trigger unnecessary diagnostic cascades, increase healthcare utilization, and undermine trust in AI-assisted clinical decision-making. Furthermore, multimodal models may introduce or amplify biases related to clinical context or training data. Recently, concerns regarding the transparency, reliability, fairness, and accountability of medical large language models were highlighted, emphasizing the need for rigorous validation and governance frameworks prior to clinical implementation [33,34]. These considerations underscore that careful evaluation of safety, bias, and ethical implications is essential before such systems can be responsibly integrated into radiological workflows.

## 5. Conclusions

While GPT-4o and Claude Sonnet 3.5 achieved excellent sensitivity for detecting brain metastases, their poor specificity, frequent hallucinations, and limited spatial accuracy preclude clinical application at present. With further refinement, domain-specific training, and careful evaluation of the interaction between textual and visual input, vLLMs may nonetheless evolve into valuable assistive tools in radiology workflows.

## Figures and Tables

**Table 1:** Demographic and Clinical Characteristics of the Study Cohort.

**Variable**		
**Mean age; SD**	58.8 (y); SD ± 12.7	
**Sex**	female 45/77 (58.4%); male 32/77 (41.6%)	
**Primary tumor**	Patients without brain metastases (46/77; 59.7%)	Patients with brain metastases (31/77; 40.3%)
**Melanoma**	15/46 (32.6%)	9/31 (29.0%)
**NSCLC**	15/46 (32.6%)	8/31 (25.8%)
**Breast Cancer**	10/46 (21.7%)	7/31 (22.6%)
**RCC**	6/46 (13.0%)	7/31 (22.6%)

Abbreviations: NSCLC—Non-Small Cell Lung Cancer; RCC—Renal Cell Carcinoma; SD—standard deviation.

**Table 2:** Objective evaluation of AI-generated reports by GPT-4o and Sonnet 3.5 across multiple interpretation categories.

Category	Accuracy GPT-4o (%)	Accuracy Sonnet 3.5 (%)	*p*-Value
Sensitivity	100% (92.9%, 100%) [50/50]	100% (92.9%, 100%) [50/50]	1.00
Specificity	8% (3.2%, 18.8%) [4/50]	4% (1.1%, 13.5%) [2/50]	0.625
Diagnostic Accuracy	54% [54/100]	52% [52/100]	0.625

Sequence Correctly Detected	100% (96.3%, 100%) [100/100]	93% (86.3%, 96.6%) [93/100]	0.0156
Too Many Lesions Detected *	12% (5.6%, 23.8%) [6/50]	12% (5.6%, 23.8%) [6/50]	1.00
Localization Correctly Detected *	70% (56.2%, 80.9%) [35/50]	72% (58.3%, 82.5%) [36/50]	1.00
Side Correctly Detected *	62% (48.2%, 74.1%) [31/50]	76% (62.6%, 85.7%) [38/50]	0.189

Abbreviations: n/a—not applicable. (): = 95% Wilson Confidence Interval. * only cases with metastases were considered.

## Data Availability

The data presented in this study are available on request from the corresponding author due to privacy restrictions.

## Supplementary Materials

The following supporting information can be downloaded at: https://www.mdpi.com/article/10.3390/diagnostics16050749/s1, Figure S1: Comparison of two aiRR based on identical MRI findings.

## Abbreviations

The following abbreviations are used in this manuscript:

AI	Artificial Intelligence
aiRR	AI-generated Radiology Report
C	Claude Sonnet 3.5 (Anthropic)
CNN	Convolutional Neural Network
FLAIR	Fluid Attenuated Inversion Recovery
G	GPT-4o (OpenAI)
JPEG	Joint Photographic Experts Group
LLM	Large Language Model
NSCLC	Non-Small Cell Lung Cancer
ViTs	Vision Transformers
vLLM	Visual Large Language Model
