# Supplementary material for: Opportunities and Challenges of Visual Large Language Models in Imaging Diagnostics: Lessons from Brain Metastasis Detection in Clinical MRI
Source: Diagnostics (Basel). 2026 Mar 3;16(5):749. doi: 10.3390/diagnostics16050749 (PMC12984547; doi:10.3390/diagnostics16050749)
Supplement: Supplementary file 1 [file diagnostics-16-00749-s001.zip › Supplementary Figure 1 Caption.pdf]

Supplementary Figure S1: Comparison of two aiRR based on identical MRI findings. Both reports pertain to the same case, shown in Figure 2. **a)**, generated using GPT-4o. **b)**, generated by Sonnet 3.5. Both reports correctly detect the presence of a metastasis, but vary in the level of detail. While GPT-4o indicates the correct side but the wrong anatomic region, Sonnet 3.5 indicates the correct anatomic region but the wrong side.
