# Supplementary figures and images for: Opportunities and Challenges of Visual Large Language Models in Imaging Diagnostics: Lessons from Brain Metastasis Detection in Clinical MRI
Source: Diagnostics (Basel). 2026 Mar 3;16(5):749. doi: 10.3390/diagnostics16050749 (PMC12984547; doi:10.3390/diagnostics16050749)

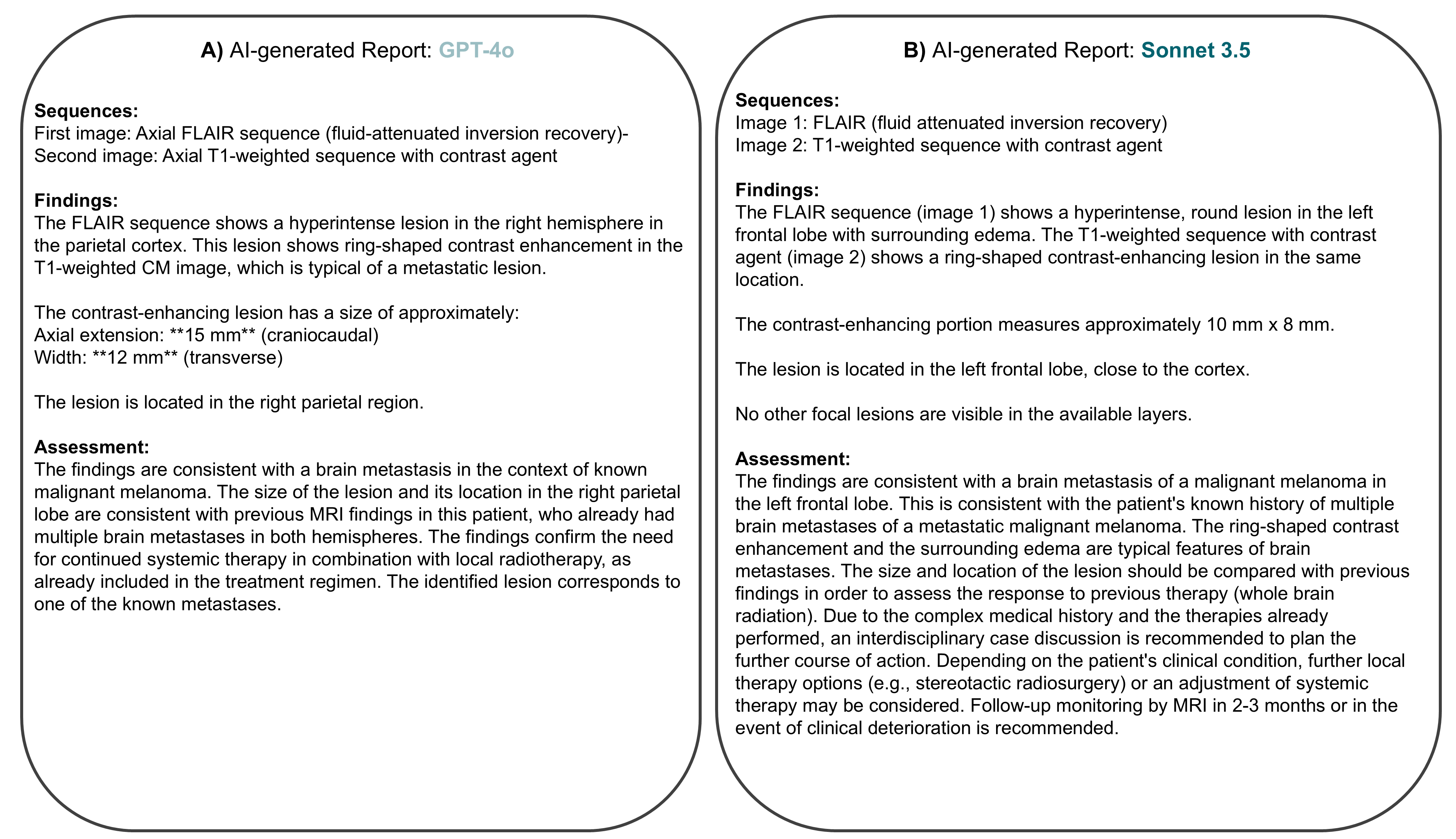

Supplement: Supplementary file 1 [file diagnostics-16-00749-s001.zip › Supplementary Figure 1.TIF]
